# Chinese consumers’ willingness to get a COVID-19 vaccine and willingness to pay for it

**DOI:** 10.1371/journal.pone.0250112

**Published:** 2021-05-04

**Authors:** Wen Qin, Erpeng Wang, Zhengyu Ni

**Affiliations:** School of Economics and Management, Nanjing Tech University, Nanjing, China; University of Florida, UNITED STATES

## Abstract

A COVID-19 vaccine is the key to beating the virus, and effective vaccines are going to be available in the near future. It is urgent to estimate the acceptability of COVID-19 vaccines and their value to individuals, in order to develop an effective public vaccination strategy. Based on a survey of 1,188 randomly selected respondents in China, we analyzed Chinese consumers’ willingness to get a COVID-19 vaccine and their willingness to pay for it. We find that 79.41% of the respondents are willing to get vaccinated in China, and the average amount that they’re willing to pay for a COVID-19 vaccine shot is 130.45 *yuan*. However, though the elderly are at higher risk of infection and the disease could be fatal for them, they are less willing to get the vaccine and not willing to pay as much for the shot. Subsidies and health communication concerning COVID-19 vaccines should be provided in order to expand vaccination coverage.

## Introduction

COVID-19, a serious respiratory illness, broke out in December 2019 and has quickly spread all over the whole world. The spread of the virus from person to person has been confirmed, and it has been found that the virus has higher transmissibility and is more likely to evolve into a pandemic than SARS-CoV [1]. By30 August 2020, more than 25 million cases of COVID-19 have been reported globally, with the U.S., Brazil and India leading the count, according to data from Johns Hopkins University [2]. The pandemic has led to severe global socioeconomic disruption and serious consumer stockpiling behavior [3]. By August 2020, no specific antiviral treatment has been proved effective [4]. For the general public, the best way of prevention is to wear masks and isolate themselves at home to avoid infection [5]. In this situation, multiple investments are made to conduct research into the development of COVID-19 vaccines which is the key to beating the virus. Although no vaccine has completed clinical trials, there are multiple attempts in progress. It is expected that effective vaccines would be available in the near future.

While scientists around the world are rushing to create a COVID-19 vaccine, health communication experts say they need to start to lay the groundwork for acceptance now. Efficient investments in health protection require valid estimates of the public’s willingness to forgo consumption for diminished probabilities of disease [6]. The effectiveness of a vaccine also depends on the vaccination rate, because considering how contagious COVID-19 is, experts estimate that typically 70% to 90% of the population needs to be immune to achieve herd immunity [7]. Whether the public is willing to get vaccinated will determine whether they can reach herd immunity. In France, 26% of the respondents said they wouldn’t get a coronavirus vaccine, and a survey of 1,056 American people in mid-May showed that just 50% of Americans planned to get a COVID-19 vaccine [8]. Now there is little knowledge about vaccination behavior [9]. Few studies using contingent valuation surveys have estimated the willingness to pay for vaccines [9, 10]. Considering that differences across racial or ethnic groups may exacerbate racial or ethnic disparities in vaccination [11], it is important to understand Chinese people’s vaccination behavior in order to make an effective vaccination strategy.

China’s immunization program has some characteristics that differ from those of other countries [12]. About 11 vaccines including those against hepatitis B and MMR (measles, mumps, rubella) are free for all children under 14 years of age, and these government-purchased vaccines are called Category 1 vaccines. In contrast, Category 2 vaccines, like the HPV vaccine and the flu vaccine, are paid by individuals. Take the flu vaccine for example. It is not currently included in the national Expanded Program on Immunization (EPI) in China. In Shanghai, a flu shot costs 53 to 136 *yuan* ($7 to $20) [13]. For the COVID-19 vaccine, there is still not a flat list price in China. Consumers in Wuhan need to pay 234 *yuan* ($35.83) for each shot, while it is free for high-risk groups at Guangdong, Zhejiang and Shandong provinces. The officials of China’s health departments claimed the vaccine price would depend on its scale of use and China will ensure the COVID-19 vaccines be affordable to all Chinese residents [14].

The objective of this study is to estimate the acceptability of a COVID-19 vaccine and its value to individuals, in order to develop an effective public vaccination strategy in China. Based on a survey of 1,188 randomly selected respondents in China, we estimate residents’ willingness to get vaccinated and their willingness to pay for a COVID-19 vaccine shot. Understanding consumers’ vaccination behavior could provide critical information for policy makers to help them identify possible barriers and develop strategies. This study also makes an important contribution to the few existing health economics studies on COVID-19 vaccination behavior.

## Data collection

### Sample

**Fig 1:**
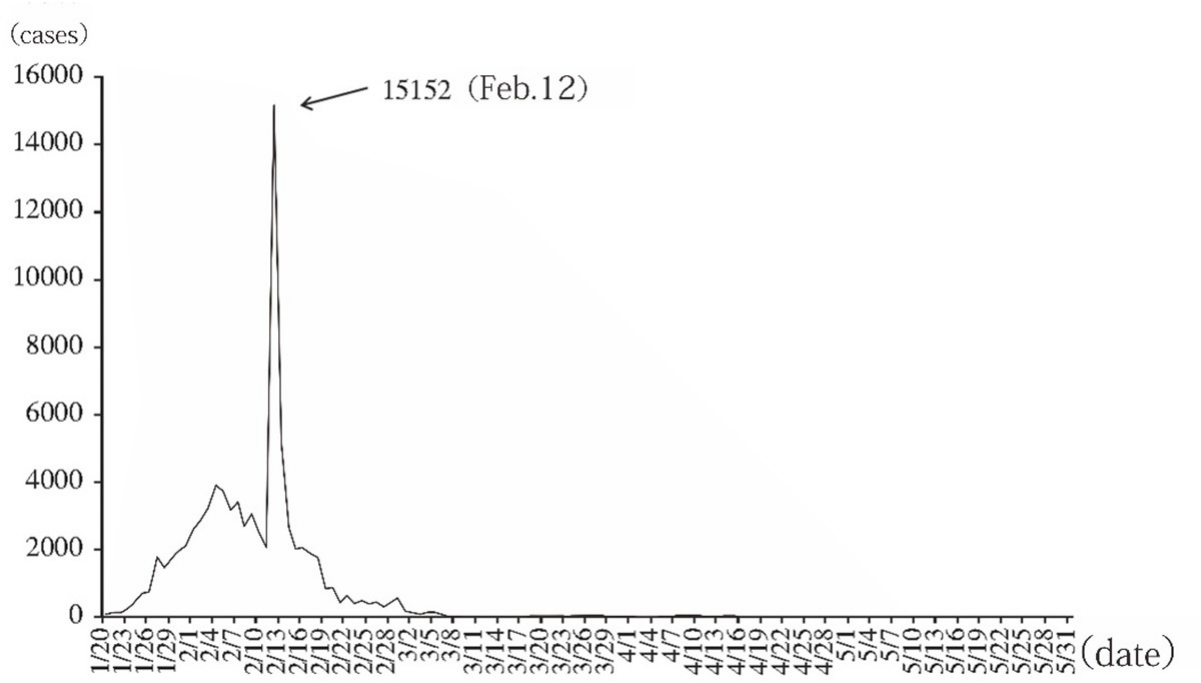
Daily figure of newly confirmed cases on the Chinese mainland [16]. Note: On February 12, newly confirmed cases reached 15,152 (including 13,332 cumulative clinically diagnosed cases in Hubei).

The online survey was conducted by a professional marketing research company (SO JUMP) from 11 to 13 March 2020 in China. Respondents all received daily briefings on novel coronavirus cases from March 10 to March 12. On March 10, 15 new cases of confirmed infections, 33 new cases of suspected infections, and 11 new deaths (10 in Hubei province) were reported on the Chinese mainland. During that time, the government locked down Wuhan and encouraged people to stay indoors to contain the spread of the virus. Around 42,600 health care professionals from all over China joined forces with the local medical workers to treat the patients and combating the epidemic [15]. Health care workers also provided residents with anti-epidemic preventive care, disinfection, publicity, and education. Through painstaking efforts and tremendous sacrifice, China has succeeded in controlling the spread of COVID-19. There were very few newly confirmed cases on the Chinese mainland between March 10 and March 12 (Fig 1), according to the white paper titled “Fighting COVID-19: China in Action”, which was issued by China’s State Council Information Office. The study was deemed exempt from requiring human subjects’ approval by the Institutional Review Board of Nanjing Tech University. The survey of this study contained no identifier items, such as name, address, etc. At the beginning of the questionnaire, we sought the consent of the participants. Only if they agreed, they would complete the questionnaire. And when filling out the questionnaire, they could skip any questions at any time. Our research does not involve any medical records or archived samples at all.

Samples, randomly selected from a huge sample database (with 2.6 million samples), were from several representative provinces/cities. Hubei province, the epicenter of the coronavirus outbreak in China, is the most seriously affected. There were 67,781 cumulative cases of COVID-19 on March 10. Guangdong and Zhejiang provinces, with a large population, are the second worst pandemic-stricken regions, with 1,356 and 1,215 cumulative cases on March 10. Jiangsu, Hebei and Shaanxi provinces represent the areas with less risk of infection. Beijing is the political and cultural center of China. Shanghai is representative of global financial centers. The sample provinces/cities together account for 33.87% of the national population, and take 90% of all the cumulative cases on March 10 (there were 80,778 cumulative cases on March 10 in China). Table 1 reports their population and the daily figure of COVID-19 cases.

**Table 1:** The population and daily figure of COVID-19 cases in sample areas.

	**Population**	**Proportion in the national population**	**Newly confirmed cases on March 10**	**Newly confirmed cases on March 11**	**Newly confirmed cases on March 12**	**The cumulative cases on March 10**
Hubei	5,927	4.23%	8	5	4	67,781
Guangdong	11,521	8.23%	0	1(suspected case)	0	1,356
Zhejiang	5,850	4.18%	0	1(asymptomatic case)	0	1,215
Jiangsu	8,070	5.76%	0	0	0	631
Beijing	2,154	1.54%	0	1	0	435
Shanghai	2,428	1.73%	0	0	0	344
Hebei	7,592	5.42%	0	0	0	318
Shaanxi	3,876	2.77%	0	0	0	245
Total	47,418	33.87%	8	6	4	72,325

### Survey design

The survey was divided into three sections. The first part included questions about respondents’ characteristics, such as age, gender, monthly household income, educational level and perception of COVID-19 risk. The second part asked about consumers’ willingness to get a COVID-19 vaccine. Respondents were directly asked, *“Do you plan to get a coronavirus vaccine when one is possible*?*”*

The third section was to elicit respondents’ willingness to pay for vaccines. Contingent valuation method (CVM) is one of popular stated preference methods to assess the value of nonmarket goods [17, 18]. CVM usually includes the continuous methods and the discrete method [19]. The payment card approach is one of the continuous methods of CVM, which is prevalent in the current literature [20–22]. Therefore, we used the payment card method to elicit respondents’ willingness to pay for vaccines. We asked respondents to assume that the price for a flu vaccine is 50 *yuan* when the mortality rate of flu is 0.1% [23], then requested them to pick the maximum acceptable price for a coronavirus vaccine if the mortality rate of COVID-19 is 0.4% [24]. Hence, we set the choices of price in the payment card to include 7 ranges: 50–75 *yuan*, 75–100 *yuan*, 100–125 *yuan*, 125–150 *yuan*, 150–200 *yuan*, 200–250 *yuan*, 250–300 *yuan*, and above 300 *yuan*.

### Econometric model

A binary logit regression model was used to study the willingness to get vaccinated. If the respondent is willing, the dependent variable equals 1, and 0 if not. The binary logit regression model is the main technique to tackle such a data structure. Assuming that willingness to get vaccinated can be modeled as a linear function,

$$y_{i}=X_{i}^{\prime }.\beta +\varepsilon_{i}$$ (1)

Where *y_i_* is whether willing to get vaccinated and *X_i_* is a vector of demographic variables, *ε_i_* is the random error.

The interval regression was used to study the willingness to pay for vaccines, because the WTP from the payment card method consists of intervals and censoring observation. The interval regression is a generalized Tobit model [25], and the differences among censored, truncated, and interval data have been discussed by Cameron and Trivedi [26]. If the value for the *j*th individual is somewhere in the interval [*y*_1*j*_, *y*_2*j*_], the known boundaries of WTP, as well as the chosen range indicating the underlying maximum WTP for vaccination, are taken into the consideration of the interval-censored model. Since the interval data boundaries are already known, true WTP is assumed to locate in regions (−∞,*y*_1_),(*y*_1_,*y*_2_],…(*y_J_*,∞). Assuming a latent variable WTP* indicates the true WTP by individual *i*:

$${WTP}^{*}=x_{i}^{\prime }\beta +u_{i},\;and\;{WTP}^{*}|x∼Normal(x^{\prime }\beta ,\sigma^{2})$$ (2)

## Results and discussion

### Willingness to get vaccinated

Removing the respondents with any missing responses, we collected 1,188 valid samples (42% on 11th, 30% on 12th, 28% on 13th) from Beijing, Shanghai, Hubei, Guangdong, Zhejiang, Jiangsu, Hebei and Shaanxi. Table 2 reports the demographics of the samples. About 43.85% of the survey respondents were female. 34.27% of the respondents were over than 35 years old. 141 respondents claimed there were COVID-19 cases in their communities, 40 from Hubei province, 52 from Guangdong province, 19 from Hebei province, 8 from Jiangsu province, 7 from Zhejiang province, 6 from Shaanxi province, 5 from Beijing, and 4 from Shanghai.

**Table 2:** Descriptive statistics.

**Sample Variable**	**Pooled sample (N = 1188)**	**Willing to get vaccinated**	**WTP**
Gender:			
Male	56.15%	78.42%	129.92
Female	43.85%	80.20%	131.14
Age:			
≤ 24 years old	21.46%	83.64%	139.95
25–34 years old	44.28%	81.67%	147.58
35–44 years old	22.56%	78.65%	117.42
45–54 years old	9.18%	63.56%	112.67
≥55 years old	2.53%	68.75%	111.93
Education level:			
≤ 12 years	21.92%	76.98%	125.47
> 12 years	78.08%	80.10%	138.63
Personal monthly income:			
< 2000 yuan	14.73%	76.56%	127.72
2000–3999 yuan	14.23%	80.66%	127.23
4000–5999 yuan	21.21%	76.92%	121.13
6000–7999 yuan	15.91%	80.40%	140.78
8000–9999 yuan	13.72%	84.80%	144.14
10,000–14,999 yuan	14.14%	80.68%	154.84
≥ 15,000 yuan	6.06%	75.00%	150.66
Children under 12 years old:			
No	42.67%	74.49%	127.47
Yes	57.33%	83.08%	132.67
Total	100%	79.41%%	130.45

Table 2 reports the statistics of respondents’ willingness to get vaccinated. The average rate of willingness to get a vaccine is 79.41%, while 87.96% of the respondents in Hubei province report they are willing to get a vaccine against COVID-19. If the vaccine is effective and can ensure immunity, mass vaccination may help achieve herd immunity according experts’ estimation [7]. Interestingly, respondents in Beijing and Shanghai are least willing to get vaccinated (73.24% and 65.63%, respectively), which is not consistent with the general view that income is positively associated with the demand for vaccines [27]. There is a significant difference in the rate of willingness to get vaccinated among different age groups. Based on currently available information and clinical expertise, older adults and people of any age who have serious underlying medical conditions might be at higher risk of developing severe illnesses from COVID-19. However, the rate of willingness to get vaccinated is low in the elderly group.

### Willingness to pay for vaccines against COVID-19

**Fig 2:**
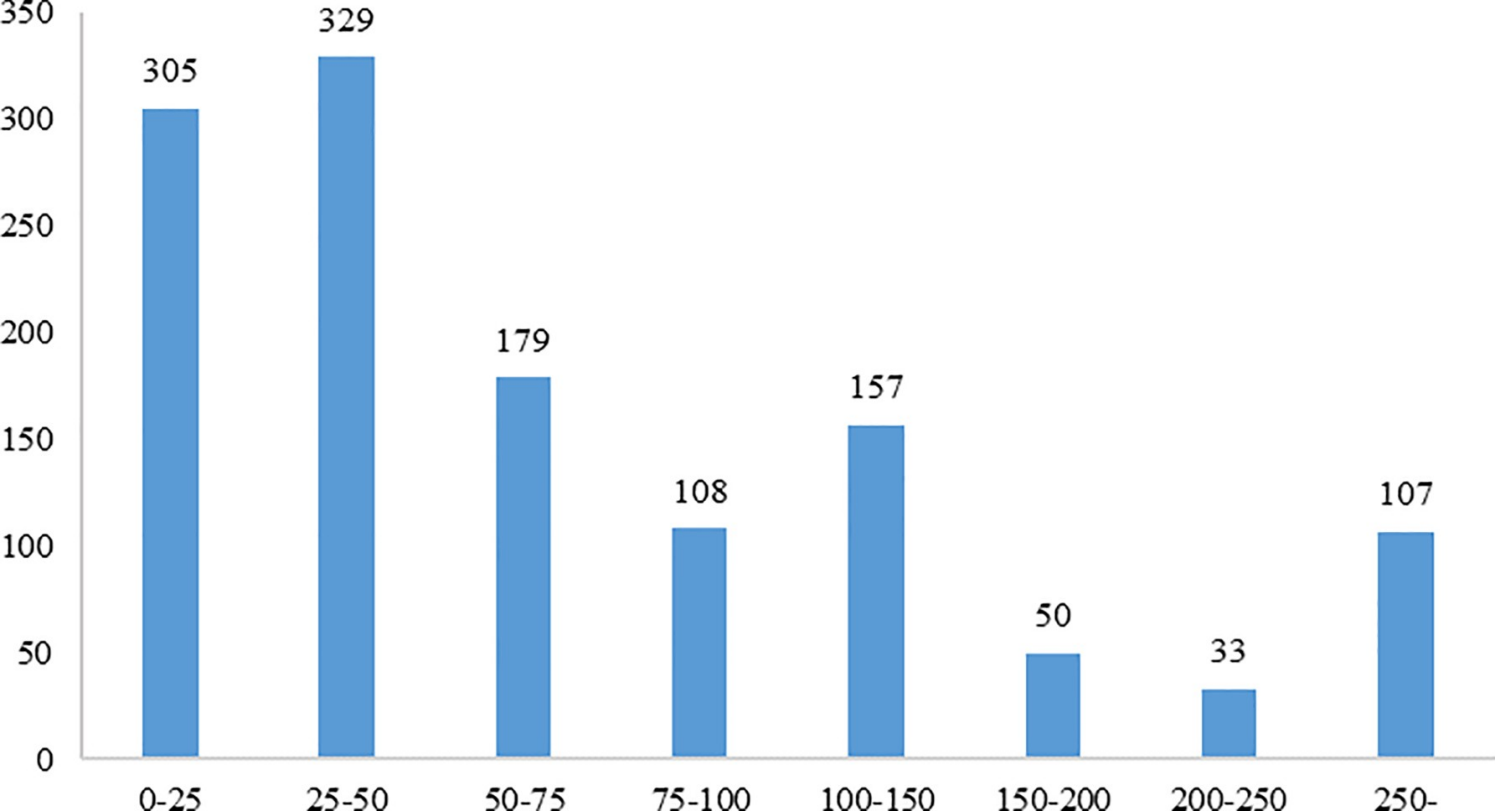
The frequency of WTP for a COVID-19 vaccine shot.

Fig 2 reports the statistics of respondents’ willingness to pay for a COVID-19 vaccine shot. The average amount that respondents are willing to pay for a vaccine is 130.45 *yuan* (the WTP value for the up-open interval is equal to the sum of the lower boundary and the half distance of the neighboring interval). Compared to the price of a flu vaccine, 50% of the respondents pay an amount of less than 100 *yuan* for a vaccine shot. This may be because the number of COVID-19 cases continues to fall in China during March 2020.

### Who are willing to get a COVID-19 vaccine

Using a binary logit regression model, we identify variables that have a statistically significant association with the probability of willingness to get a vaccine. The dependent variable willingness to get vaccinated equals 1 if the respondent is willing to get vaccinated and 0 if not. Table 3 reports the regression results.

**Table 3:** Regression results.

**Variables**	**Willingness to get vaccinated**		**WTP for a vaccine shot**
	**Coef.**	**Odds Ratio**	**Coef.**
Female	-0.163	0.849	0.605
	-0.262		-0.906
Age (≤ 24 years old)	0.615**	1.85	9.242
	-0.017		-0.269
Age (35–44 years old)	-0.313*	0.732	-28.96***
	-0.099		0
Age (45–54 years old)	-0.752***	0.472	-32.75***
	-0.001		-0.001
Age (≥55 years old)	-0.606	0.545	-27.59
	-0.137		-0.112
Educational level	-0.011	0.989	-3.895
	-0.953		-0.561
Income (2000–3999 *yuan*)	0.620**	1.859	12.76
	-0.03		-0.202
Income (4000–5999 *yuan*)	0.415	1.514	6.849
	-0.134		-0.496
Income (6000–7999 *yuan*)	0.544*	1.723	26.29**
	-0.07		-0.014
Income (8000–9999 *yuan*)	0.862***	2.369	33.55***
	-0.01		-0.003
Income (10,000–14,999 *yuan*)	0.495	1.64	43.05***
	-0.127		0
Income (≥ 15,000 yuan)	0.241	1.272	39.00***
	-0.523		-0.006
Children under 12 years old	0.552***	1.737	-1.723
	0		-0.761
*AIC*	1,269.90		4,689.20
*BIC*	1,341.90		4,763

Results show that age is associated with the willingness to get vaccinated. Taking those from 25–34 years of age as a baseline, younger respondents are more likely to be willing to get a vaccine against COVID-19. However, respondents from 35–44 years and 45–54 years of age are less willing to be vaccinated, even though the COVID-19 guidance claims that older adults seem to be at higher risk of developing more serious complications from COVID-19 illness. Also, those having a high personal monthly income (8000–9999 *yuan*) are generally more willing to get vaccines, compared to the respondents with low incomes. Some personal monthly income variables are positive but not significant. Besides, respondents that have children under 12 years old are more willing to get vaccinated.

Furthermore, we use an interval regression model to study the hypothetical WTP for vaccination. The dependent variable is the interval of WTP. As expected, WTP for vaccination is positively associated with income. The elderly are not willing to pay as much for a COVID-19 vaccine shot. The families with children are less willingness to make a payment than others, because most vaccines are free for children in China.

## Conclusions and discussion

COVID-19 (coronavirus disease 2019), an ongoing global pandemic, is the most extensive to afflict humanity in a century. Although no COVID-19 vaccine has been accomplished, the Phase 3 clinical trial published earlier in the fourth week of July 2020 is very encouraging and promising [28]. Designing an effective vaccination strategy demands the identification of consumers’ willingness to get vaccinated and their willingness to pay for a COVID-19 vaccine.

This paper provides important information for policy makers to help them make an effective vaccination strategy. Firstly, this study shows that the proportion of those willing to get vaccinated in China is 79.41%, which is quite higher than that in France and the U.S. It means vaccination coverage in different countries is quite divergent. Secondly, although the average amount respondents are willing to pay for vaccines in China is 130.45 *yuan*, more than half of respondents are not willing to pay more than 100 *yuan* for a COVID-19 vaccine shot. The regression results also show that income also matters. Excessively high price of the COVID-19 vaccine may deter a substantial share of the at-risk people with low incomes from getting vaccinated. Partial subsidies would be needed to raise the vaccination rate. Thirdly, although the COVID-19 guidance claims that older adults seem to be at higher risk than the young, they are less willing to get vaccinated, and are not willing to pay as much for a COVID-19 vaccine shot.

Although our samples were from several representative provinces/cities, randomly selected from a huge sample database (with 2.6 million samples), it may not be an accurate representation of China’s population. This is because the people with a low educational level and the elderly are less likely to response to the online survey. To draw a more robust conclusion, future research can conduct similar studies covering more provinces of China, especially the rural areas. Future studies can also analyze the impacts of population density, trust in government and risk perception on consumers’ willingness to get vaccinated.

## Supporting information

S1 Data (XLSX)
